# P-786. Comparison of Length of Stay and Hospitalization Costs Among Patients with Complicated Urinary Tract Infection including Acute Pyelonephritis by Antibiotic Hospital Discharge Treatment Strategy

**DOI:** 10.1093/ofid/ofaf695.997

**Published:** 2026-01-11

**Authors:** Thomas Lodise, Amy G Edgecomb, Fanny S Mitrani-Gold, Jeffrey J Ellis, Alin Kalayjian, Lindsey Parker, Benjamin Chastek, Timothy Barnes, Aaron Lucas

**Affiliations:** Albany College of Pharmacy and Health Sciences, Stratton, VA, United States, Stratton, VA; GSK, Collegeville, PA, United States, Collegeville, Pennsylvania; GlaxoSmithKline plc., Deerfield , IL; GSK, Collegeville, Pennsylvania; GSK, Collegeville, PA, United States, Collegeville, Pennsylvania; GSK, Collegeville, PA, United States, Collegeville, Pennsylvania; Optum, Eden Prairie, Minnesota; Optum, Eden Prairie, MN, United States, Eden Prairie, Minnesota; Pittsburgh VA Medical Center; Pittsburgh, PA, United States, Pittsburgh, Pennsylvania

## Abstract

**Background:**

Complicated urinary tract infections (cUTIs) and acute pyelonephritis (AP) often require hospitalization and intravenous (IV) antibiotics. Patients may complete treatment in hospital (IV complete), be discharged with outpatient parenteral antibiotic therapy (IV-to-OPAT) in settings such as home health or skilled nursing facilities, or transition to oral antibiotics (IV-to-PO). Understanding differences in length of stay (LOS) and costs between these strategies is critical for optimizing care and resource use.

**Figure:**
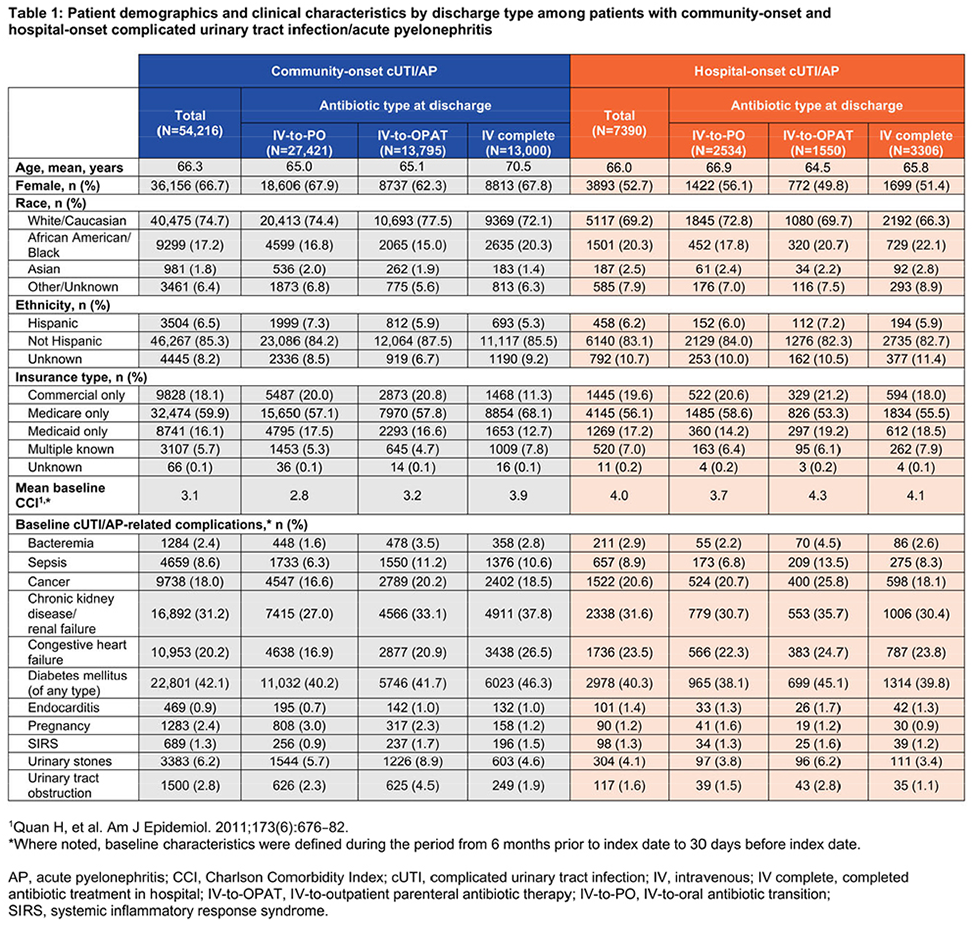

**Figure:**
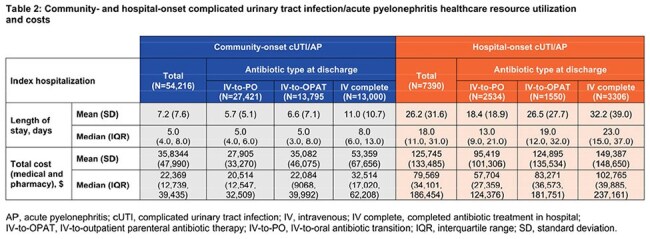

**Methods:**

This retrospective cohort study used Optum’s de-identified electronic health record-linked-claims data (Optum Market Clarity) between 10/01/2015 and 09/30/2023, for hospitalized adults with cUTI/AP receiving IV antibiotics. Hospital admission was defined as the index date. Patients were categorized by the timing of urine culture collection – community-onset (-/+2 days index) versus hospital-onset (≥ +3 days index) – and stratified by Charlson Comorbidity Index score (CCI: 0, 1─2, 3─4, ≥ 5). LOS and costs were compared across treatment strategies (IV-to-PO, IV-to-OPAT, IV complete) and CCI risk groups.

**Figure:**
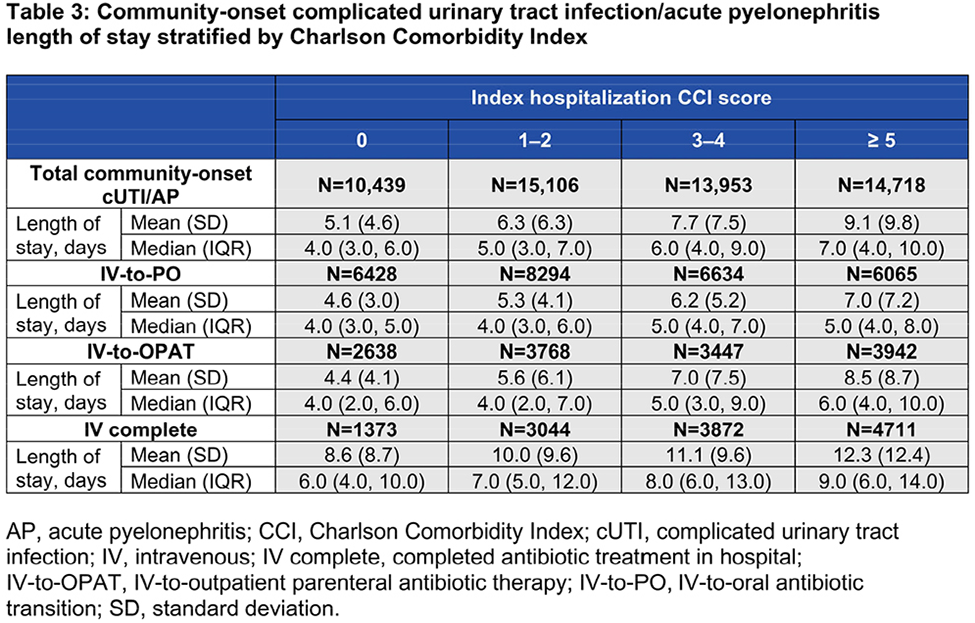

**Figure:**
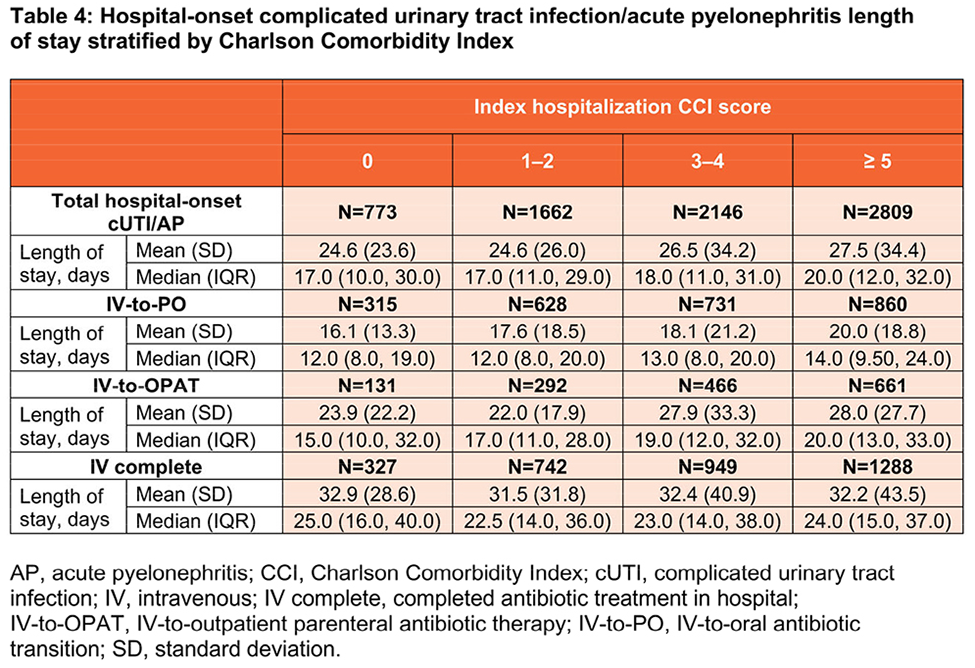

**Results:**

Among 54,216 community-onset cases, 50.6% were IV-to-PO, 25.4% IV-to-OPAT, and 24% IV complete. Among 7390 hospital-onset cases, 34.3% were IV-to-PO, 21% IV-to-OPAT, and 44.7% IV complete. Demographics and clinical characteristics are described in Table 1. For community-onset, IV-to-PO had the shortest LOS (mean 5.7 days) and lowest mean cost ($27,905) versus IV-to-OPAT (6.6 days; $35,082) and IV complete (11 days; $53,359). LOS increased with comorbidity; median LOS for community-onset ranged from 4 days (CCI 0) to 7 days (CCI ≥ 5; Tables 2 and 3). Hospital-onset cases showed similar patterns: IV-to-PO (18.4 days; $95,419), IV-to-OPAT (26.5 days; $124,895), IV complete (32.0 days; $149,387). Median LOS for hospital-onset ranged from 17 days (CCI 0) to 20 days (CCI ≥ 5; Tables 2 and 4).

**Conclusion:**

Patients that transitioned from IV-to-PO antibiotics had both shorter LOS and lower costs than those managed solely on IV antibiotics (either in OPAT or hospital settings). Higher LOS in IV complete and IV-to-OPAT groups may be influenced by disease complexity, frailty and non-treatment related factors.

Funding: GSK study 221141.

**Disclosures:**

Thomas Lodise, Jr., PharmD, PhD, GSK: Advisor/Consultant Amy G. Edgecomb, PharmD, MPH, GSK: Employee|GSK: Stocks/Bonds (Public Company) Fanny S. Mitrani-Gold, MPH, GSK: Employee|GSK: Stocks/Bonds (Public Company) Jeffrey J. Ellis, PharmD, MS, GSK: Employee|GSK: Stocks/Bonds (Public Company) Alin Kalayjian, PharmD, MS, MBA, GSK: Previous employment|Penumbra, Inc.: Employee|Penumbra, Inc.: Stocks/Bonds (Public Company) Lindsey Parker, PharmD, GSK: Employee|GSK: Stocks/Bonds (Public Company) Benjamin Chastek, MS, Optum (UnitedHealth Group): Stocks/Bonds (Public Company) Timothy Barnes, MHI, MBA, Optum (UnitedHealth Group): Employee|Optum (UnitedHealth Group): Stocks/Bonds (Public Company) Aaron Lucas, MD, GSK: Advisor/Consultant|Optum (UnitedHealth Group): Advisor/Consultant

